# 1,3,5-triazines inhibit osteosarcoma and avert lung metastasis in a patient-derived orthotopic xenograft mouse model with favorable pharmacokinetics

**DOI:** 10.22038/IJBMS.2022.62705.13873

**Published:** 2022-03

**Authors:** Qing Su, Baolin Xu, Zhoubin Tian, Ziling Gong

**Affiliations:** 1 Department of Orthopedic Oncology, Yantai Shan Hospital, Yantai, 264003, China; 2 Department of Orthopedics, The Second Affiliated Hospital Zhejiang University School of Medicine, Hangzhou, 310006, China; 3 Departments of Joint Surgery, Shandong Provincial Hospital Affiliated to Shandong First Medical University, Jinan, 250021, China; 4 Department of Orthopaedic Surgery, Shanghai Jiao Tong University Affiliated Sixth People’s Hospital, Shanghai, 200233, China

**Keywords:** 1, 3, 5-triazine, Apoptosis, mTOR, Osteosarcoma, PI3K

## Abstract

**Objective(s):**

Osteosarcoma is a major solid malignant tumor of bone, possessing significant burden on healthcare due to non-availability of specific anticancer agents. The current study was conducted to identify novel 1,3,5-triazine derivatives against osteosarcoma.

**Materials and Methods:**

The compounds were synthesized in a straight-forward two-step reaction and subsequently tested against PI3K and mTOR kinase and anticancer activity against osteosarcoma cells (MG-63, U2-OS, and Saos-2). The effect of the most potent compound was evaluated on apoptosis and cell phase of Saos-2 cells. The pharmacological activity was further established in the patient-derived orthotopic xenograft (PDOX) mouse model.

**Results:**

The developed compounds 8 (a-f) showed significant inhibitory activities against PI3K, mTOR, and OS cells. Among the tested series, compound **8a** showed highly potent PI3K/mTOR inhibitory activity with significant anticancer activity against Saos-2 cells compared with Imatinib as standard. It also induces apoptosis and causes G2/M arrest in Saos-2 cells. Compound **8a** significantly improved body weight, reduced tumor volume, and inhibited lung metastasis in athymic nude mice in a PDOX mouse model. It also showed optimal pharmacokinetic parameters in SD rats.

**Conclusion:**

In summary, 1,3,5-triazine analogs were identified as new PI3K/mTOR inhibitors against osteosarcoma.

## Introduction

Cancer is the most dreadful disease ever known to humankind. Despite various advances in therapeutics and diagnostics, still, the management of cancer is troublesome (1). Among the types of cancer affecting humans, osteosarcoma is the most common solid malignant tumor of bones (2, 3). Despite advances in chemotherapy, prognosis and survivability of patients are not very encouraging. Various studies suggested that in the past 40 years, the overall survivability of non-metastatic osteosarcoma patients remained sluggish (4).Thus Thus, the development of novel targeted therapies is urgently needed to fill the void. 

Imatinib mesylate (Gleevec) is a potent tyrosine kinase anticancer molecule widely used against bcr-abl-positive chronic myeloid leukemia (5-7). Additionally, it showed a significant effect on the bone cells where it targets MCF receptors and induces apoptosis of mature osteoblast (8). Imatinib significantly inhibited the proliferation of osteosarcoma cells by arresting cell-cycle and provoking caspase-dependent apoptosis (9-11). The above studies showed the significant potential of Imatinib against osteosarcoma. 

Studies have found that phosphatidylinositol-3-kinase (PI3K)/Akt and the mammalian target of rapamycin (mTOR) are aberrantly activated in osteosarcoma (12). This pathway is critically involved in cell proliferation, growth, cell size, metabolism, and motility. Drugs such as Duvelisib, Copanlisib, and Idelalisib showed potent inhibition of the PI3K/mTOR pathway and were approved clinically against many tumors, including osteosarcoma (13). Thus, discovery of new drugs to inhibit or modulate PI3K/mTOR offers superior benefits against osteocarcinoma. 

**Figure 1:**
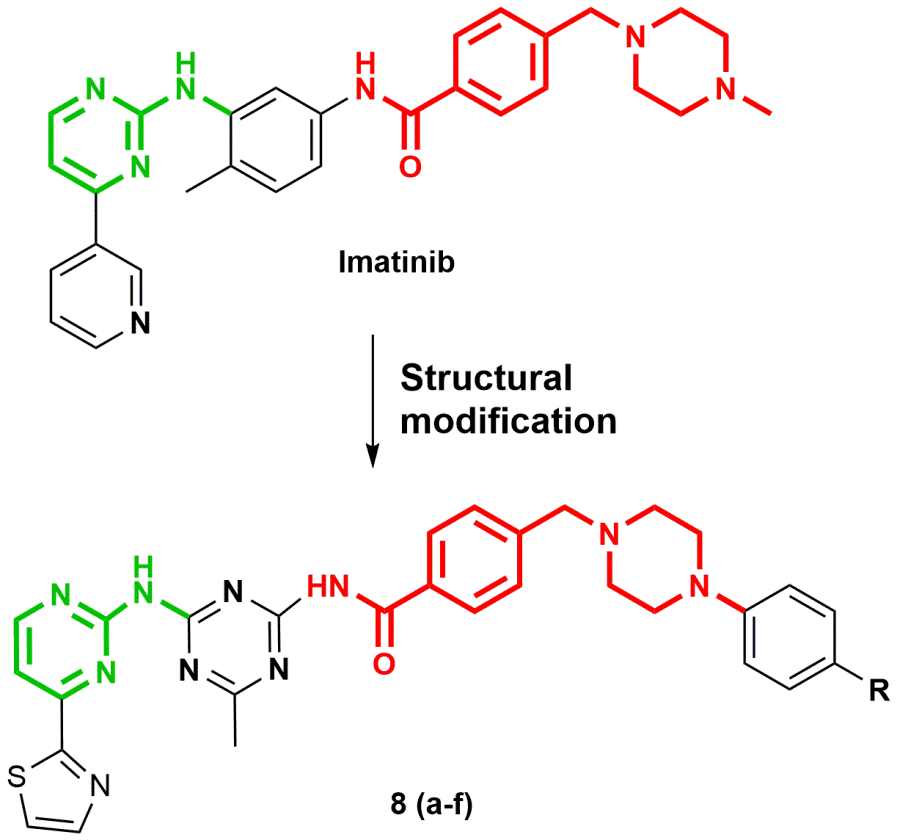
Design of target compounds inspired by Imatinib

Almost 80 % of inhibitors used in the treatment of cancers belong to the class of heterocyclic small molecules (14). These molecules are very highly sought over others because of ease of synthesis, intrinsic versatility, and unique physicochemical properties that can be fine-tuned in the light of biological activity, toxicity, and pharmacokinetic activity to obtain molecularly targeted agents. 1,3,5-triazine, a highly active pharmacophore showed a diverse array of biological activity against disease-causing pathogenic organisms, such as bacteria (15–20), fungus (21–23)malaria (24–29), HIV (30), HIV (30), diabetes (15), and cystic fibrosis (31). Much of the work has also been documented on the discovery of anticancer agents from 1,3,5-triazine scaffold (32). It inhibits numerous kinases for anticancer activity, for instance, Cyclin-dependent kinase (CDK)(33), epidermal growth factor receptor tyrosine kinase (EGFR-TK) (34), and phosphatidylinositol 3-kinase/AKT/mammalian target of rapamycin (PI3K/Akt/mTOR)(35, 36). Moreover, various 1,3,5-triazines were proven as effective antileukemic agents (37). In our previous study, we developed a novel series of 1,3,5-triazine nicotinohydrazide as potent osteosarcoma agents via inhibition of CDK (38). Therefore, in continuation of our anti-osteosarcoma research program, the present study enumerates the anti-osteosarcoma activity of 1,3,5-triazine analogs inspired by Imatinib and elucidation of its mechanism of action (Figure 1). We have also determined the effect of our designed compound on lung metastases of osteosarcoma because patients affected by osteosarcoma present poor prognosis due to lung metastasis of the disease (39). 

## Materials and Methods

***Chemistry***

The molecules were synthesized as per the earlier reported procedure and they were characterized with the help of melting point which is found in agreement with the earlier reported melting points (40). 

***PI3K and mTOR kinase inhibition assay***

The inhibitory activity of developed molecules against PI3K and mTOR was identified using luminescent kinase assay, and Lance Ultra assay from Promega, USA, respectively as per our earlier reported procedure (38).

***Pharmacological activity***

*Cells*

The human osteosarcoma cancer cell lines MG-63, U2-OS, and Saos-2 were purchased from CBSIBCB of the Chinese Academy of Sciences (Shanghai, China) 

*Cellular antiproliferation assay using cell counting kit (CCK-8)*

The CCK-8 assay was used in the current study to analyze the effect of compounds on cellular proliferation. The transfected cells were seeded in a 96-well plate, and to this CCK-8 (20 µl) was added. The enzyme microplate reader at 450 nm was used to record the absorbance. The inhibitory effect on cellular proliferation was obtained as per the given formula: Inhibitory activity (%) = (1 - A_test_/A_control_) × 100 %.

*Cell cycle analysis*

The effect of compound 8a (0, 10, 20, and 30 µM) was investigated on the cell cycle of Saos-2 cells as per our earlier reported procedure using a FACSCalibur flow cytometer with CellQuest V.3.3 software (BD Biosciences, USA) (38).

***Flow cytometric apoptosis assay***

The effect of compound **8a** (0, 10, 20, and 30 µM) on apoptosis was investigated on Saos-2 cells as per our earlier reported procedure using a FACSCalibur flow cytometer with CellQuest V.3.3 software (BD Biosciences, USA) (38).

***In vivo pharmacological activity***

*Surgical orthotopic implantation (SOI) for the establishment of the osteosarcoma PDOX model*

The PDOX model in Athymic nu/nu nude mice was established according to our earlier reported procedure (38). The tumor tissue for implantation was obtained from a 10-year old OS patient with written informed consent.

*Treatment protocol*

The mice were arbitrarily categorized into four dissimilar groups (n=6), and the treatment schedule lasted for 14 days as follows. The compound **8a** was dissolved in the solution of PEG400/Tween 80/Saline solution at 10/10/80 % in volume, respectively prior to intraperitoneal administration. 

Group 1: Untreated

Groups 2, 3, and 4 received compound **8a** (2, 5, and 10 mg/kg, respectively, IP, daily)

The control animals received the vehicle only.

The length and width of the tumor along with the bodyweight of mice were recorded.

The tumor volume was calculated with the following formula:

Tumor volume (mm^3^)= length (mm) × width (mm) × height (mm))⁄2

*Examination of lung histopathology*

At the end of the experiment, the lung tissues were harvested and fixed in formalin (10%). The tissues were further entrenched in paraffin, sectioned using a microtome into 5-μm thickness. The resulting sections were dyed using hematoxylin-eosin (HE). 

*Pharmacokinetic assessment*

The compound **8a** was administered to female SD rats in a single dose of 1 and 5 mg/kg using intra-venomous and per-oral route in the vehicle. The blood samples from the rats were collected in a timely manner starting from 2 min to 24 hr. The serum was extracted from the blood-aliquots for the estimation of various pharmacokinetic parameters (T_1/2, _T_max, _C_max_, AUC_0-INF_) developed using WinNonlin software using LC-MS/MS (Applied Biosystem, USA).

***Statistical analysis***

One-way analysis of variance (ANOVA) was conducted with the Tukey test for *post-hoc* analysis. The *P*-values of 0.05 or less were considered statistically significant.

## Results

***Chemistry***

The earlier reported procedure by Guan and Jiang (40) was adopted to synthesize the target compounds and the authenticity of compounds was ascertained by using elemental analysis which was found in ± 0.4% of the theoretical values. 

***Kinase inhibition study***

The inhibitory activity of designed analogs was tested against PI3K and mTOR and Imatinib as a positive control, Table 1. It has been found that all compounds **8a-f** showed significant to moderate IC_50_ against both kinases in the nanomolar range. The compounds showed a diverse variety of inhibitory profiles against both kinases. Compound **8a **exhibited outstanding inhibitory activity among the tested derivatives against PI3K (IC_50_ = 786 nM) and mTOR (IC_50_= 345 nM). In the next instance, on replacing chloro with bromo (**8b**), a slight reduction in kinase inhibitory activity was observed with further decrease in the case of compound **8c**, containing *p*-methyl. The compound **8d **having the *p*-nitro group demonstrated moderate improvement in inhibitory activity against the tested kinase. However, the removal of nitro and the insertion of hydroxy (OH) or methoxy (OCH_3_) do not significantly influence the inhibitory activity. These analogs showed mild to mostly minor activity against PI3K and mTOR. However, none of the synthesized compounds showed better activity than Imatinib (IC_50_ value of 312 nM and 214 nM against PI3K, and mTOR kinase, respectively).

***Anticancer activity***

Impressed by the exceptional kinase inhibitory profile of the designed analogs, it is worthwhile to test its inhibitory activity against various osteosarcoma cells (K562, KU812, and Saos-2), Table 1. It has been shown that compound **8a** inhibits the survival of both K562 and KU812. However, it showed potent inhibitory activity against Saos-2 (IC_50_= 210 nM) compared with Imatinib (IC_50_= 332 nM) as standard. The anti-proliferative activity of compound**s 8b** and **8c** was found significantly reduced against all tested cell lines. The inhibitory potency was found significantly increased in the case of compound **8d**, whereas compounds **8e** and **8f** showed reduction in inhibitory potency. On closely monitoring the above results, it was inferred that developed compounds showed an approximately similar inhibition pattern against the tested kinases and osteosarcoma cells. Compound **8a** was the most potent analog among the tested series in both *in vitro* experiments, while compound **8f** showed the least activity.

Impressed by the strong anticancer effect of compound **8a**, we intend to perform the mechanistic analysis underlying its anticancer effect.

***Effect on compound 8a cell cycle of Saos-2 cells***

**Figure 2:**
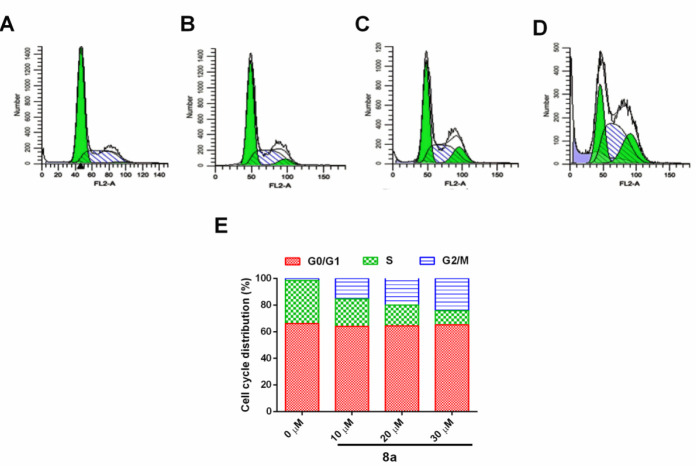
Effect of compound 8a on the cell cycle progression of Saos-2 cells, where: (A) control, (B) 10 µM, (C) 20 µM, (D) 30 µM, and (E) cell cycle division percentage

As shown in Figure 2, the compound 8a treated group showed that S-phase cells were found almost similar with increase in G2/M phase cells. These results indicated that compound** 8a** causes G2/M phase arrest.

***Effect of compound 8a on the apoptosis of Saos-2 cells***

**Figure 3:**
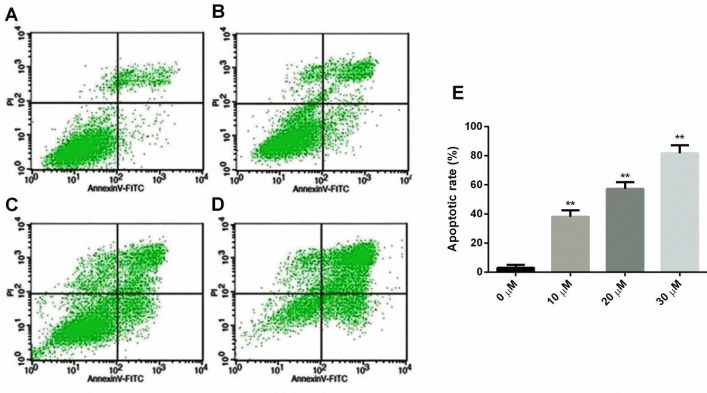
Effect of compound 8a on cell apoptosis of Saos-2 cells determined by using flow cytometry, where: A) control, (B) 10 µM, (C) 20 µM, (D) 30 µM, and (E) comparative bar-graph of flow cytometry analysis estimating the percentage of cell apoptosis rate. Data were shown as mean ± SEM **P<0.01 vs control

As shown in Figure 3, compound **8a** dose-dependently increases apoptosis of Saos-2 cells in Annexin V/PI analysis as evidenced by an increase in the percent of apoptosis rate. 

***In vivo*** **activity**

**Figure 4:**
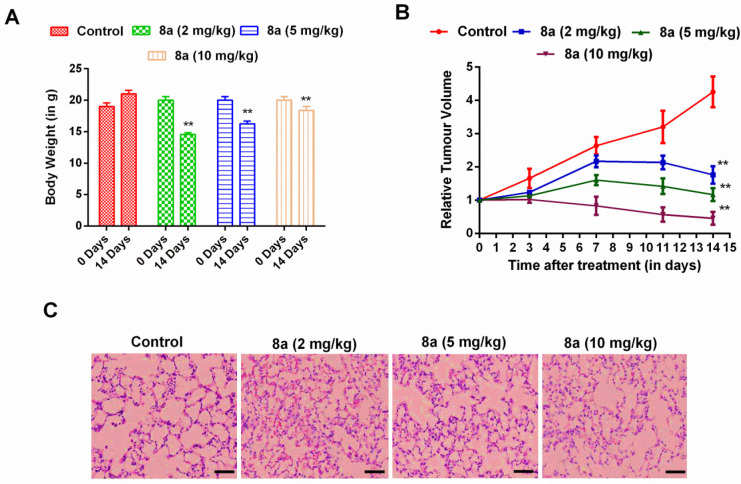
Anticancer effect of 8a on PDOX mouse model. (A) mice body weight, (B) relative tumor volume all through the study schedule, and (C) lung histopathology of mice corresponding to different treatment groups. Data are shown as mean ± SEM **P<0.01 vs control

In continuation of the above experiments, in the next study, we have studied the bioactivity of compound **8a** in the patient-derived orthotopic xenograft (PDOX) mouse model. Initially, the pharmacological benefit of molecule **8a** was assessed based on two parameters, viz., body weight and relative tumor volume. As shown in Figure 4, mice treated with compound **8a** showed improvement in body weight in comparison with control. The tumor volume was found significantly reduced in the **8a** treated group at the end of the study. Thus, on the basis of the above results, it has been suggested that compound **8a** showed excellent anti-cancer activity against patient-derived osteosarcoma cells. 

***Effect on lung metastasis***

As shown in Figure 4C, the control group showed well-structured alveoli with no alterations. Compound **8a** in low doses (2 mg/kg) does not have a significant effect on the metastasis of the lung as evident by disordered lung architecture, ruptured alveoli with some necrotic portion, and increased permeability due to interstitial hemorrhages. However, in medium dose at 5 mg/kg, mice revealed less necrotic lesions and minimum interstitial hemorrhages. In the high dose of compound 8a (10 mg/kg), the lung architecture of treated mice was found significantly restored close to normal with reduced disease lesions. Thus, it could be suggested that compound **8a** significantly ameliorated lung metastasis of osteocarcinoma in a dose-dependent manner. 

***Pharmacokinetic assessment***

As shown in Table 2, Compound **8a** showed excellent pharmacokinetics with t_max_ and t_1/2_ of 160 min and 210 min, respectively in *p.o.* route. On the other hand, in i.v. route, compound 8a attained peak plasma level at 8 min with C_max_ of 8126 suggesting that it distributed swiftly across the body-compartments. The AUC of compound **8a **was found significantly acceptable in both tested dosing routes. Results of the above pharmacokinetic study confirm that compound **8a** is well-tolerated with optimal pharmacokinetics.

**Scheme 1:**
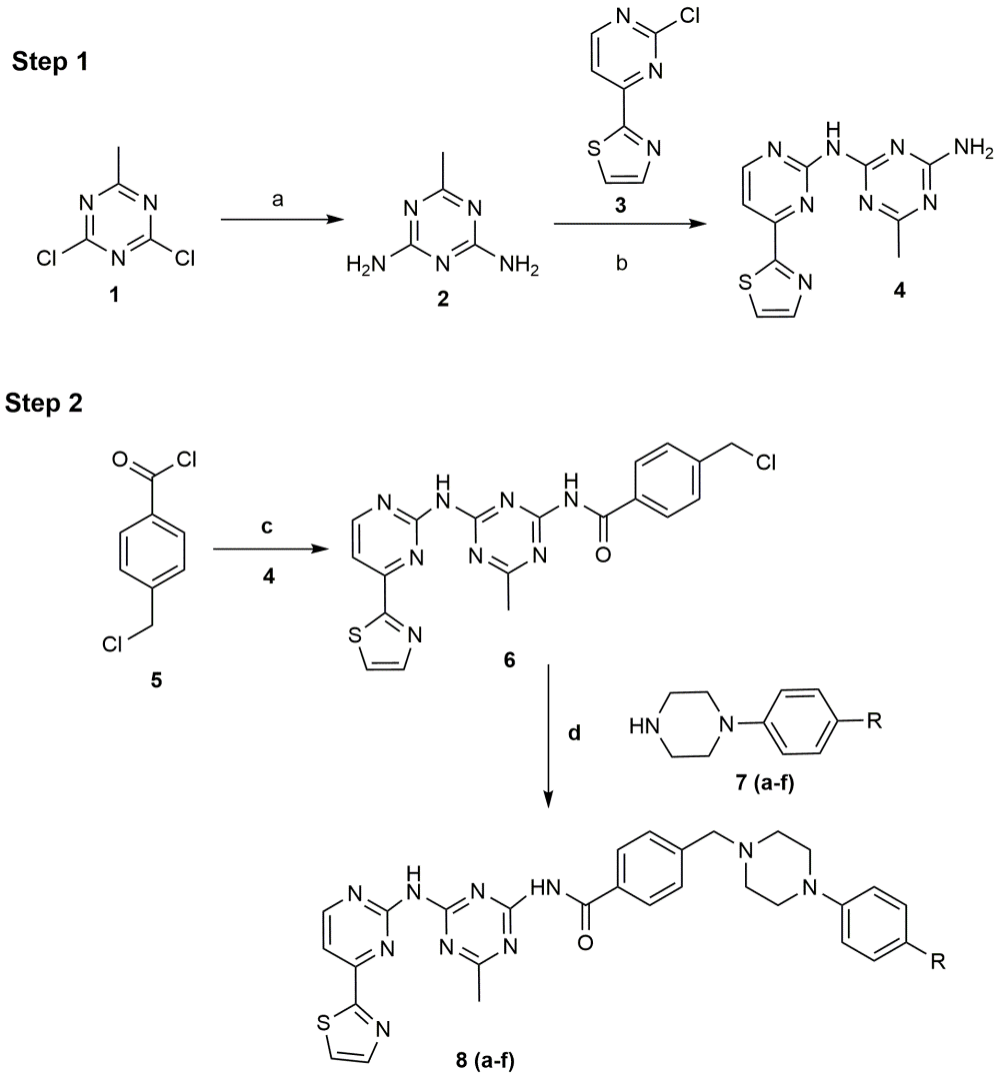
Reagents and conditions: a) Liq. NH_3_, 40-45 °C, NaHCO_3_, heat, 2–3 hr, b) NaOBu-t/THF 10 °C to RT, 2 hr, c) THF-TEA, 0 °C, 3 hr, d) reflux, 3–5 hr

**Table 1:** **I**nhibitory activity of designed compounds against PI3K, mTOR, and anticancer activity against osteosarcoma cells

Compound	R	Kinase inhibition IC_50 _(nM)		Cell growth inhibition IC_50_ (nM)		
				K562	KU812	Saos-2
		PI3K	mTOR			
**8a**	4-Cl	786	345	620	722	210
**8b**	4-Br	885	389	730	830	268
**8c**	4-CH_3_	967	490	900	1032	530
**8d**	4-NO_2_	905	414	822	950	340
**8e**	4-OH	987	456	887	1140	478
**8f**	4-OCH_3_	1247	834	1200	1234	905
**Imatinib**		312	214	410	165	332

**Table 2:** Pharmacokinetic parameter of compound 8a in SD rats

**Parameter**	**PO (5 mg/kg)**	**IV (1 mg/kg)**
T ½ (min)	210	45
T _max_ (min)	160	8
C _max_ (ng/ml)	410	8126
AUC _0- INF_ (hr*ng/mL)	297326	61201

## Discussion

Osteosarcoma (OS) is a malignant tumor of bone that originates in the mesenchymal tissue and is responsible for 20% of all cases of primary malignant bone tumors in the world. In the early 70 sec, surgery was the only option to treat OS which later used chemotherapy as an adjuvant treatment to eliminate the formation of metastases that would not be possible to remove by surgery alone (2, 42). However, resistance to chemotherapy has compromised the clinical utility of current therapeutic modalities and decreased the overall prognosis of the disease (4). In our present study, we have successfully demonstrated the anti-osteosarcoma activity of 1,3,5-triazine derivatives as potent inhibitors of the PI3K/mTOR pathway. 

Cell growth is mainly dependent upon the highly conserved biological process known as the cell cycle. Under abnormal circumstances, it has been found abruptly de-regulated and serves as a characteristic hallmark of cancer (43). Various cell-cycle-specific inhibitors were used as a primary therapeutic modality or in combination with other drugs against cancer (44, 45). Therefore, initially, we have enumerated the effect of the most potent inhibitor (**8a**) on the cell cycle of Saos-2 cells. Results of the study suggest that compound **8a** causes G2/M phase arrest. Apoptosis is a process which is termed programmed cell death response to maintain tissue homeostasis. Studies have shown that apoptosis is found aberrantly unbridled in various cancers, including osteosarcoma (45). Results suggest that compound **8a** causes a dose-dependent increase of apoptosis of Saos-2 cells. Thus, it is suggested that compound **8a** showed a robust anticancer effect against osteosarcoma cells possibly by promoting apoptosis and cell cycle arrest of the G2/M phase. Concerning the above benefit of compound **8a** against osteosarcoma, it is worthwhile to assess the pharmacological activity of **8a** in *in vivo* experiments. Therefore, we have chosen our established patient-derived orthotopic xenograft (PDOX) mouse model for the bioactivity determination of compound **8a** on various biochemical parameters. According to the American Cancer Society, body weight is considered the first noticeable symptom of the cancer effect. New molecule improves bodyweight because of the anticancer effect; whereas, relative tumor volume directly correlates with the anticancer effect of the test compound on the tumor tissues. The results suggest that compound **8a** showed excellent anti-OS activity ascertained on the basis of decreased tumor volume and increase in body weight of **8a**-treated mice at the end of the study. 

The survivability of osteosarcoma patients has been seriously jeopardized due to lung metastases (46). As per the estimate, osteosarcoma has been found metastasized to the lungs of the patients at the time of the first diagnosis (47). Surgical resection is a current first-line of therapy to treat osteosarcoma patients affected with lung metastasis followed by a chemotherapy regimen (48). Despite this, the relapse of disease is quite frequent in the majority of cases even after using various chemotherapeutic drugs. Thus, the management of osteosarcoma patients with lung metastasis is quite challenging (39). Thus, it is imperative to define the effect of compound **8a** on lung metastasis of the PDOX mice. It has been found that compound **8a** significantly ameliorated lung metastasis of osteocarcinoma via restoring the lung architecture in a dose-dependent manner. The potency of any pharmacological agent is highly dependent on its bioavailability. It needs to stay in the body in a bioactive form long enough for the expected biological events to occur. Thus, the study of any new lead’s pharmacokinetic properties is imperative in the early drug discovery process. Concerning this and encouraged by the excellent pharmacological profile of compound **8a**, lastly, we estimate its pharmacokinetics profile in SD rats. Results of the study suggested that compound **8a** is well-tolerated with optimal pharmacokinetics.

## Conclusion

In summary, a series of 1,3,5-triazine derivatives were designed and synthesized as new PI3K/mTOR inhibitors. The resulting compounds significantly attenuate the activity of both PI3K and mTOR and potently inhibit the propagation of various osteocarcinoma cells. The results of the above studies enumerated compound **8a** as a potent inhibitor of Saos-2 cells. Compound **8a** also induced apoptosis and causes G2/M phase arrest of Saos-2 cells. It also showed dose-dependent inhibition of tumor volume and increase in body weight in the patient-derived orthotopic xenograft (PDOX) mouse model. Compound **8a** significantly ameliorated lung metastasis of osteocarcinoma via restoring the lung architecture in a dose-dependent manner and showed excellent bioavailability in the pharmacokinetic assay. Collectively, compound **8a** is a capable anticancer lead for further development.

## Authors’ Contributions

QS performed experiments and formal analysis, BX performed experiments, ZT performed experiments and analyzed the data, ZG conceptualized and supervised the study. All authors approved the current version of the manuscript. 

## Compliance with Ethical Standard

The ethical committee of Shanghai Jiao Tong University Affiliated Sixth People’s Hospital approved this study (IMREC/SPU/2020/A23). All animal experiments were conducted following the experimental animal guidelines set by the National Institutes of Health Guide for the Care and Use of Laboratory Animals.

## Conflicts of Interest

The authors declare no conflicts of interest.
